# Barriers to Type 1 Diabetes Adherence in Adolescents

**DOI:** 10.3390/jcm13195669

**Published:** 2024-09-24

**Authors:** Sarah Azar, Noa Maroun Abou Jaoude, Andrzej Kędzia, Elżbieta Niechciał

**Affiliations:** Department of Pediatric Diabetes, Clinical Auxology and Obesity, Poznan University of Medical Sciences, Szpitalna Street 27/33, 60-572 Poznan, Poland; sarahazar5@gmail.com (S.A.); noa.abj5@gmail.com (N.M.A.J.); akedzia@ump.edu.pl (A.K.)

**Keywords:** type 1 diabetes, adolescents, treatment, regimen, adherence, compliance, barriers, psychological aspects, puberty, diabetes technology

## Abstract

**Background:** Adolescence is a particularly crucial period of physical, emotional, and social development and adaptation, rendering these formative years rather challenging for individuals with chronic conditions like type 1 diabetes (T1D). Despite rapid improvement in diabetes therapies, adolescents with T1D are characterized by poorer adherence to treatment regimens compared with other pediatric age groups. Insufficient adherence is strongly related to low diabetes control, increasing morbidity, and risk for premature mortality. This study aimed to provide a comprehensive overview of adolescents’ most common barriers to T1D adherence, stressing the need for a deep and comprehensive understanding of these barriers. The complexity of these barriers is underscored by the diverse factors contributing to poor T1D adherence in adolescents. **Methods:** A narrative review was conducted, surveying four databases (PubMed, Scopus, EMBASE, and Web of Science) for full-text articles in the English language published up to June 2024. All studies related to barriers to T1D adherence in adolescents were considered. The literature was selected using selection and exclusion criteria and extracted and organized using Mendeley. Exclusion criteria included studies with insufficient data and non-peer-reviewed articles. This review revealed that adolescents face numerous obstacles to T1D adherence related to psychological factors, flux in family dynamics, perceived social pressures, therapy-related factors, transitioning responsibility, risk-taking behaviors, and pubertal changes. **Conclusions:** Navigating the adaptations to the different aspects of T1D, from treatment to complications and adolescents’ personal growth, effectively requires a thorough understanding of the barriers of a treatment regimen that patients at this critical age face.

## 1. Introduction

Energy metabolism throughout the body is effectively modulated, from production and storage to utilization by insulin and glucagon. Insulin plays a vital role in the fate of macronutrients, mainly carbohydrates, to achieve optimal energy usage while regulating glucose levels within the bloodstream to a set threshold of approximately 70 mg/dL (3.9 mmol/L) [1,2]. Hyperglycemia in type 1 diabetes (T1D) is derived from the autoimmune destruction of pancreatic islet β-cells, leading to a substantial decrease and loss of the amount of insulin produced and released in the body [3]. Thus, individuals with T1D require lifelong insulin treatment to survive, either via multiple daily injections (MDI) or continuous subcutaneous insulin infusion (CSII). Proper daily management of T1D is complex and covers carbohydrate counting, adjusting insulin doses, and blood glucose monitoring, as well as how to manage physical activity, illness, or hypoglycemia [4]. 

T1D can develop at any age; however, in pediatric populations, it occurs at two noticeable peaks, with the first peak incidence of onset at age 4–6 years and the second in early puberty (10–14 years) [5]. It has been estimated that more than 1.2 million children and adolescents younger than 20 years worldwide have T1D [6]. Consequently, T1D is assumed to be the most common metabolic disorder among children and adolescents, imposing substantial pressure upon these patients in terms of lifelong adherence to therapy [7]. According to the American Diabetes Association, patients who acquire intensive insulin therapy demonstrate lower HbA1c levels and decreased macrovascular and microvascular complications. Hence, adherence to insulin therapy is one of the key elements to achieving optimal glycemic control [8]. 

However, the recommended regimen is not limited to diligent medical care but demands different factors that impact the initiation and persistence of insulin treatment. Patients must constantly check their blood glucose levels up to six times daily, adjust their insulin dosage to their carbohydrate intake for each meal or exercise, and follow a diet plan [9]. Implementing such a demanding regimen in adolescents substantially burdens their everyday lives. Priorities of enjoyment and carefree experiences are hallmarks of such an age group, which tend to divert their attention away from a strong emphasis on health and well-being. Adolescence is a phase characterized by constant change and adaptation to newfound physical and emotional experiences. During this time, adolescents are not only adjusting to their developing changes but mostly to an emerging desire for independence. Hence, navigating a chronic condition like T1D can be challenging for such young individuals striving for freedom and social acceptance [10,11].

Family dynamics, psychological impact, complexity of regimen, limited youth knowledge, and physiological hormonal changes are some of the considerations that shape the challenges to adherence in adolescents. Regarding adherence to insulin therapy, adolescents exhibit the poorest glycemic control regarding patients in other age groups [12]. In addition, even the new medical technologies with tendencies to facilitate and enhance treatment, like continuous glucose monitoring (CGM), still challenge adherence to therapy in teenagers [13].

This article aims to thoroughly review the various barriers to adherence in adolescents with T1D.

## 2. Materials and Methods

This review is narrative, and no systematic literature search was performed; each author identified and critically reviewed the most relevant papers. Inclusion criteria were clinical studies, systematic reviews, and meta-analyses discussing barriers to T1D adherence in adolescents. Exclusion criteria included non-English language papers, studies with insufficient data, non-peer-reviewed articles, duplicated, unavailable full texts, or abstract-only papers. The following electronic databases were searched for relevant full-text articles in the English language: PubMed, Scopus, EMBASE, and Web of Science. The search time was up to June 2024 using the following keywords: type 1 diabetes; adolescents; treatment; regimen, adherence, compliance, barriers; mental health; family; transition; psychological aspects; puberty; growth; insulin sensitivity; insulin resistance; diabetes technology; diabetes devices; financial; practical issues; skin complications; dermatological complications; short term complications, long term complications, social support; lifestyle. The ethical exemption was granted due to the review nature of the study, with no direct involvement of human or animal subjects. It aimed to detect the most clinically relevant papers related to the topic and provide a theoretical point of view, considered a valuable educational tool in continuing medical education.

### 2.1. Mental Health

The nurturing significance of mental health in adolescents’ development cannot be overstated. Chronic medical diseases, such as T1D, in adolescents exacerbate mental health challenges, serving as triggers for psychiatric comorbidities such as anxiety, depression, behavioral disorders, and suicide [14]. This impairs proper management of the treatment vignette of T1D in adolescents, leading to suboptimal outcomes in their condition [14]. Symptoms of depression and anxiety both show a higher prevalence in adolescents with T1D than in the general population, ranging from about 11.3% to 27.5% and 13% to 21.3%, respectively [15,16]. In a case study on 150 teenagers with T1D, around 82% of participants screened positive for one or several mental health disorders, with the most prevalent being anxiety, comprising 21.3% of this population [16]. Anxiety has been associated with higher HBA1c levels, increased fear and concern about hypoglycemia, and constant glucose monitoring, all of which impose persistent daily life stressors on adherence to treatment [15]. Hence, anxiety as a mental disorder in youth with T1D is not just exerting its profound influence on their emotional well-being and creating an unstable emotional state of overthinking. Still, it is manifesting its impact by disrupting their treatment regimen and leading to detrimental health outcomes. A case study of adolescents with T1D and their parents assessing both perspectives on the influence of comorbid conditions on health-related quality of life (HRQOL) reported that mental health is the most important comorbidity associated with a lower HRQOL [17]. Additionally, another study performed in Poland with similar assessments as the latter indicated that among 207 children with T1D, 26.6% and 31.9% screened positive for a psychiatric disorder currently or during their lifetime, respectively. These disorders included anxiety, mood disorders, disruptive behavioral disorders, and eating disorders. Patients in this group exhibited higher HBA1c levels and demonstrated limitations in treatment adherence, thereby implying that their psychiatric disorder as a hindrance to the recommended treatment [18]. A cross-sectional study on Norwegian students reported similar general well-being among patients both with and without T1D but a higher prevalence of psychiatric disorders among patients who have a high level of HBA1c [19]. Adolescents with T1D have received referrals for mental health disorders at a rate 19% higher than that of their counterparts within 12 years after diagnosis [20], emphasizing the substantial demand for routine screening for mental health disorders as part of the T1D regimen [17]. All of these studies, taken together, demonstrate the prevalence of psychological presentation in adolescents with T1D and its correlation with poorer glycemic control and higher levels of HBA1c, emphasizing its impact as a barrier to adherence.

Finally, it must be emphasized that youth with T1D have a greater risk of developing eating disorders (EDs) compared to their healthy peers [21]. Although the causes of EDs remain unclear, they seem to be associated with focusing on diet planning, carbohydrate counting, or a fear of weight gain as a result of insulin treatment. EDs are related to poor T1D control and higher rates of disease complications. Scheuing et al. investigated the clinical characteristics of 52,215 patients with T1D, 467 of whom were diagnosed with EDs. Diabetic ketoacidosis (DKA) and recurrent hospitalization were reported to be significantly more common (*p* < 0.05), and the duration of hospital stay was longer (*p* < 0.05) in individuals with T1D presenting with mental health problems compared to those without psychiatric issues [21,22].

### 2.2. Family Dynamics

Family members are inherently interconnected, influencing each other through their experiences and challenges. Facing a chronic disease plays a pivotal role in the trajectory of a family’s routine, roles, and emotional well-being, particularly when diagnosed in pediatric patients [23]. In families with adolescents having T1D, parents are in a constant state of concern and preoccupation about the various demands of the recommended daily treatment regimen, consistency, and acceptance of adherence in patients at such an age [24]. This family distress creates a conflict environment that is strongly established as a contributing factor to treatment impairment and suboptimal glycemic controls in adolescents with T1D based on several research studies [25,26,27,28].

A qualitative review carefully assessed the impact of parenting style specifically and communication on the overall quality of life and treatment of adolescents with T1D [29]. In accordance, an earlier longitudinal study over the course of four years documented that parent adopting a rigid, controlling, disciplinarian approach towards adolescents with T1D exacerbate the challenges and poorer outcomes associated with treatment [25]. An additional study in Belgium yielded comparable results on adherence in line with the previous study, with higher HBA1c levels, mainly because of maternal intrusive handling of T1D in youth [26]. Moreover, a study of 120 adolescents and their caregivers that aimed to investigate the relationship between family functioning, youth behavioral issues, and HBA1c levels reported an 11.8% glycemic variability attributed to family functioning, with adolescents revealing higher HBA1c levels associated with a controlling parenting style towards T1D daily tasks and overall management [27]. This overbearing parental behavior can burden youth in addition to their chronic condition, resulting in resentment toward adherence.

Parent’s capacity to manage their own behaviors and control their emotions and thoughts is essential when coping with a chronic disease [28]. A study conducted by the University of Utah showed that weaker parental self-control when dealing with T1D in adolescents has been reported to decrease adherence notably, with adolescents also showing higher HBA1c levels when self-control was decreased only in fathers at the time of study but not mothers, particularly longitudinally [28]. Another contributing factor to non-adherence in youth with T1D is family structure, where households with single parents noted less compliance to treatment, as reported by a study of 165 adolescents in China [30], consistent with a study carried out in the United States [31].

Finally, being a parent also affected by T1D might have two-sided effects on adolescents’ diabetes control. On the one hand, such parent has a more excellent knowledge of the disease and their own experience, which might help them understand the complexity of T1D management. Moreover, treatment adherence can become more manageable when having a parent with T1D because psychologically, adolescents would not feel different or even as a burden. The regimen would also be considered more simplified, with an adult person in the same household having to deal with all the steps daily. On the other hand, parents with T1D, when having a child affected by the same disease, might be resistant to diabetes education because, in their opinion, they have sufficient knowledge or experience, and they might not be keen to improve their understanding. Therefore, adolescents may rely on parental experience, which is not always in line with doctors’ recommendations. As a consequence, diabetes control in adolescents could be negatively affected.

### 2.3. Transitioning Treatment Responsibility

Adolescence marks a transitional period into self-exploration and the emergence of more independent decisions and behaviors. Hence, the burden of shifting treatment regimen responsibility in T1D from parents to youth is part of the challenging development of such patients with chronic conditions [32,33,34,35]. A clinical trial reported that parents with increased involvement in diabetes care and increased levels of conflict resulted in decreased psychosocial well-being in youth without negatively affecting glycemic controls [32]. Another study reported that decreased parental engagement in handling T1D in adolescents resulted in better glycemic outcomes in the short term but increased HBA1c levels later on [33]. However, early and premature adolescent independence and decreased parental involvement in this matter disrupt proper treatment and lead to poorer metabolic control. On the other hand, the impact of prolonged parental control over T1D in youth was also mentioned, noting the resulting family discrepancies and disagreements that worsen treatment outcomes [34,35]. Glycemic variability was also evident in research on adolescents and their parents because of extended parental involvement in diabetes tasks [35]. Based on all the presented studies, there are different points of view on the degree of youth independence and parental contribution in managing T1D in youth because it varies between patients according to their level of development, growth, maturity, and understanding of the situation. Insight is critical because a chronic condition needs daily interventions and follow-up, highlighting the importance of assessing an adolescent’s knowledge before transitioning to diabetes care.

### 2.4. Social Pressure

Society can act as a hindrance or nurturing experience for adolescents, but either way, it has manifested itself as a definitive element in their daily life. Adolescents become more self-conscious about differences because social acceptance, fitting in, and being “normal” as perceived by society become constant desires they seek [36]. In turn, this social pressure applies to a greater extent in adolescents with chronic diseases such as T1D because of the potential differences in behavior. These different daily regimen tasks are required to be performed frequently in school in front of their peers in case of T1D, exacerbating increased vulnerability to bullying in such patients [37]. This bullying and social exclusion, particularly in schools, are attributed to the constraints imposed on students with chronic conditions that restrict them from participating in social activities along with their classmates, each according to their condition and how the activity affects them and their treatment regimen [38]. Hence, research shows that bullying among students with chronic conditions decreases their overall quality of life and leads to poorer compliance and health outcomes [37], emphasizing the influence of peer pressure on such students. In addition, a research study [39] on older adolescents with T1D interpreted the roles of society toward their condition as protective, indifferent, and offensive. The latter category demonstrated the unjust and mockery treatment peers at such ages treat adolescents with T1D, mainly because of their lack of insight and understanding towards the situation. They use unhealthy foods as teasing or exclusion and joke about comparing the treatment regimen and daily insulin injections to drug consumption. Adolescents with T1D struggle to cope with this daily stress and embarrassment and, thus, purposefully neglect diabetes care in front of their peers, even if this results in hypoglycemic or hyperglycemic events [39]. Another study on adolescents with T1D in a Taiwanese school reported results in line with the “offensive role [37]” just described. Students constantly felt different from their peers, so their main concern was fitting in and avoiding unwanted attention, which they received while performing the required tasks for diabetes care. Hence, they resorted to symptom changes that would alarm them of fluctuating glycemic levels instead of constant monitoring in school. These students with T1D also intentionally neglected compliance in certain situations, like parties, to blend in and enjoy themselves like the rest of their peers [40]. In almost all the presented studies, social pressure was a strong determinant of adherence because most adolescents with T1D prioritized social acceptance over their T1D management daily tasks in social settings.

### 2.5. Risk-Taking Behavior

Adolescence is often thought to be the age period characterized by experimentation with risky behaviors, such as alcohol, tobacco, or drug use, which may negatively interfere with engagement in T1D care. Although the prevalence of alcohol intake seems to be lower in adolescents with T1D compared to their healthy peers, still alcohol use is a considerable issue affecting T1D management. In the study conducted among Polish adolescents with T1D aged between 15 and 18 years of age, around 82.8% of participants admitted that they had consumed alcohol at least once during their lives, while 71.7% and 47.5% of individuals reported alcohol intake in the last 12 months and in the previous 30 days, respectively [41]. Alcohol use in adolescents with T1D decreases gluconeogenesis and glycogenolysis. Therefore, it increases the risk of severe health consequences, including hypoglycemia and diabetic ketoacidosis (DKA) [42]. Additionally, drinking alcohol impairs hypoglycemia unawareness because symptoms of its use, for example, confusion, blurred vision, and unsteady gait, might mimic the signs of low glucose concentration [43]. The rate of alcohol-related hospitalizations is the highest in children aged 14–17 years old compared to other age groups [44]. Therefore, young patients should be aware of this risk, routinely screened for alcohol consumption, and be counseled if appropriate. When it comes to drug use, cannabis intake increases the risk of DKA more than twice in adults with T1D [45,46], and some available data shows an identical trend in adolescents with T1D [47]. Meanwhile, smoking is an independent risk factor for cardiovascular complications [42].

### 2.6. Pubertal Changes and Their Effects on Insulin Sensitivity

During puberty, activation of the hypothalamic-pituitary-gonadal axis triggers neuroendocrine changes that drive the physiological transformations of the body to achieve sexual maturity [48]. Hence, these physiological adaptations likely contribute to insulin resistance (IR) during puberty in patients both with and without diabetes, exacerbating glycemic control issues in adolescents with T1D [49]. Studying pubertal changes and considering their effects on the body’s physiologic development and chronic conditions has been previously questioned and studied, highlighting puberty as a fundamental determinant of these changes [49,50,51,52,53].

In children, transient IR appears to be strongly related to pubertal hormones. According to a study that involved tracking for approximately 2 +/− 0.6 years of insulin sensitivity level in a group of 60 healthy children at the onset of puberty (Tanner stage I), a 32% reduction in insulin sensitivity was observed by reaching Tanner stage III [50]. Similarly, in the study using the hyperinsulinemic-euglycemic clamp technique to assess insulin sensitivity in children with T1D and their healthy peers in different stages of puberty (Tanner stage I and II to V), individuals from both groups in mid-puberty experienced a 25 to 30% decrease in insulin sensitivity compared to prepubertal children and adults [49]. Then, age-related changes in IR seem to be lowest in children at Tanner stage I while reaching their highest point midway through maturation (Tanner stage III) [49]. Moreover, some evidence suggests that female adolescents at all Tanner stages might develop higher IR than males at the same age [54]. An increased IR observed during puberty is mainly associated with elevated insulin-like growth factor 1 (IGF-1) levels, as demonstrated by a longitudinal study involving 342 healthy adolescents [51]. Hence, this hormonal change is regulated by growth hormone (GH) [51], which is produced twice as much during puberty compared to childhood or adulthood [49,50,51,55]. However, adolescents with T1D experienced marked GH secretion but decreased IGF-1 levels. Impaired hepatic IGF-1 production results from insulinopenia and insulin resistance, which is responsible for negative feedback and causes an increase in GH concentration [56,57,58].

To elucidate this multi-organ IR in T1D adolescents, a four-phase hyperinsulinemic-euglycemic clamp study was conducted on 35 adolescents with T1D and 22 healthy adolescents to measure insulin’s action on target tissues, along with glucose and glycerol isotope tracers [52]. This enabled investigating metabolic changes in peripheral, adipose, and hepatic tissue [52]. Adolescents with T1D were found to have lower insulin sensitivity across these three different tissue types compared to their healthy counterparts [52]. Regarding peripheral IR, a study on healthy volunteers suggests that elevated free fatty acids (FFAs) levels in the bloodstream alter glucose transport, leading to decreased muscle glycogen synthesis [53]. It is supported by the finding that the peak of glucose-6-phosphate, triggered by insulin, was lower when FFAs were elevated [53]. These high FFA concentrations in adolescents with T1D induce vascular dysfunction, reducing muscle blood flow and consequently compromising insulin delivery to muscle tissue [53,59]. On the other hand, it was found that hepatic endogenous glucose production (EGP), which is typically expected to decrease in response to insulin, remained constant in adolescents with T1D [52]. However, considering an alternative perspective from a different study by Hoffman et al., glucose effectiveness and sensitivity were measured to examine insulin effects in 24 healthy children between eight and 13 years old for 18 months [60]. Disturbances in hepatic insulin response during puberty were not detected, nor were substantial differences between sexes or Tanner stages [60].

It is challenging to estimate insulin sensitivity, given that endogenous insulin production is deficient in T1D, and the hyperinsulinemic-euglycemic clamps technique is complex. Hence, prediction equations may be a more simplified and less invasive alternative [61]. Nevertheless, assessing insulin sensitivity thoroughly at the pubertal stage is important. IR influences T1D microvascular and macrovascular complications [62], along with its correlation with body composition and body mass index (BMI) changes [63]. Proper regimen for this demographic group has embodied criteria of several recommendations, including intensive insulin therapy potentially requiring increased insulin doses [64,65], lifestyle adjustments like nutrition plans and physical activity [66,67,68], and recently introducing metformin to the T1D treatment plan in adolescents [69,70,71,72]. Integrating metformin, one of the medications used in type 2 diabetes, into a T1D regimen is coming to the forefront since it contributes to decreasing glycemic levels [69], consequently requiring less doses of insulin administered within the course of treatment [69,70], metformin is also associated with BMI and waist circumference reductions [70] as well as a decreased risk of developing cardiovascular complications [71]. However, further research is still needed to assess the efficacy of combining insulin and metformin [72]. Thus, achieving a comprehensive insight into the influence of pubertal changes on T1D and its treatment is essential for research and the introduction of certain medications like metformin at this age.

### 2.7. Barriers to Device Usage in T1D

The pivotal discovery of insulin changed everything. Moreover, diabetes technology has advanced remarkably over the past few decades [73]. Directing our focus on the demographic group examined in this review paper, CGM and CSII regimen usage presents evidence of improved monitoring and management of T1D, compared to MDI on many levels [74,75]. Thus, the adoption of these technologies is attributed to their ability to achieve better glycemic outcomes [76], enhanced lifestyle [77], and lower risks of developing acute [74] and long-term complications [78]. However, despite these improved overall health outcomes, patients inevitably face multifactorial difficulties when handling diabetes devices daily over an extended period. This can affect their motivation to comply with technologies and further impact the control of their chronic condition [79,80,81].

### 2.8. Financial Burden on the Family/Caregivers

Incorporating CSII and CGM into the therapy plan requires an ongoing financial commitment from family or caregivers, which also has to be considered by healthcare professionals. A worldwide survey with participants from 49 countries was conducted to analyze healthcare professionals’ perspectives on starting insulin pumps and CGM for pediatric patients [82]. Most were healthcare professionals and members of the International Society for Pediatric and Adolescent Diabetes (ISPAD) [82]. They confirmed that their decision to recommend diabetes technology to their young patients strongly depends on income and the extent of coverage obtainable by the insurance [82], given that self-monitoring blood glucose devices (SMBG) and CSII will represent the majority of the cost [83]. Even though advanced medical devices for T1D are increasingly available and recommended by healthcare professionals, a comparative study involving 29 European countries shows that reimbursement levels still vary substantially among countries [84]. The study reports that seven countries, Bulgaria, Estonia, Hungary, Croatia, Serbia, Portugal, and Romania, face reimbursement limits in CSII prescription, while two countries, Latvia and Lithuania, provide no CSII reimbursement at all [84]. These disparities in accessing technologies are observed in many children in Europe and other populations [84,85,86]. To confirm that patients’ socioeconomic status impedes the implementation of devices in their diabetes treatment and clinical outcomes, a large comparative study involving over 56,000 patients under 18 years old with T1D from the United States and Germany focused on the impact of the socioeconomic factor [87]. Children from disadvantaged socioeconomic backgrounds in both countries were less likely to manage their disease with devices, which directly translated into glycemic control worsening, indicating higher HbA1c levels [87]. Maintaining CSII in the long term is crucial to managing this chronic disease, but life-long financial burdens also accompany it. A study conducted in Western Australia noted parents’ overall satisfaction with their child’s health outcomes and motivation to continue CSII [79]. However, the considerable challenge of affording sustainability arose when the subsidized pump program ended [79]. Thus, 58% of the participating families face difficulties in securing their child’s next pump due to low income, unstable jobs, single parenthood, or special needs [79]. As demonstrated by the presented research, financial constraints strongly impact access to diabetes technology worldwide, creating substantial obstacles to sustainably using these life-improving devices.

### 2.9. Ethnic Differences in Barriers to T1D

What is more, ethnic origin may negatively influence adherence to T1D treatment; in particular, ethnic minority youth are at greater risk for poorer glycemic control. A longitudinal study in East London observed HbA1c variations during the first six months post-diagnosis in a diverse group of T1D patients under 19 years old, including White, mixed ethnicity, Black, African-Somali, Bangladeshi, and Asian (non-Bangladeshi) [88]. Firstly, the study demonstrated that at diagnosis, HbA1c levels were higher in all ethnic minority groups compared to White patients, with Bangladeshis and Asians having the highest mean differences [88]. Secondly, despite an overall decrease in HbA1c levels being observed in all ethnicities throughout the six months, the scope of this difference remained the same [88]. The study highlighted the disparities in glycemic control among different ethnicities, emphasizing the necessity of early involvement to reduce these inequities [88]. On the other hand, interviews with 39 Hispanic and Black patients with T1D in New York revealed that both ethnicities encountered obstacles in managing T1D; however, the specific area of difficulty was unique to each group [89]. While Hispanic young adults faced dietary adaptation challenges, Black patients reported problems related to the healthcare system and on a social level [89]. Another large pediatric cohort study [90] examined the differences in diabetes management among White, Hispanic, and Black patients with T1D. Whites and Hispanics demonstrated better HBA1c control and less diabetic ketoacidosis and severe hypoglycemic levels than black patients. White adolescents were observed to be more likely than Hispanic and Black adolescents to use insulin pumps. These results were observed after various factor adjustments, particularly for socioeconomic status, indicating other barriers that still need to be researched [90]. Additionally, a study conducted in Denmark [91] aimed to compare the metabolic status of children and adolescents with T1D among ethnic groups in the country with that among Danish pediatric patients. It was reported that ethnic minorities had higher HBA1c levels than Danish patients, exacerbated by the challenges that these groups face. To elaborate, the lack of comprehensive knowledge and education to manage their health leads to inadequate healthcare support among ethnic groups, where no significant difference was observed in glycemic levels within themselves [91].

### 2.10. Routine Tasks and Practical Issues

T1D patients using devices to manage their glucose levels must thoroughly and consistently adhere to the multiple tasks associated with wearing them to ensure optimal performance [92,93]. Therefore, to elaborate on the scope of this regimen, various commitments required by these demanding devices entail daily tasks such as carrying supplies, delivering boluses, and monitoring CGM results [92]. Additionally, changing the CSII infusion sets every two to three days is strongly advised to prevent device-related technical issues, as will be elaborated upon subsequently [93]. Furthermore, CGM sensors should be charged weekly, while monthly duties include sending data to the provider, adjusting and evaluating treatment plans with medical professionals, and reordering supplies as required [92]. These routine engagements may indeed be complicated, confirming that using insulin pumps is specifically characterized and defined by the patients as requiring exceeding efforts [80]. Besides the regular device-related tasks that require demanding effort by adolescents, another study that involved adults and families of T1D children in New Zealand demonstrated that device malfunctions constitute 38% of the potential adverse effects associated with CSII [94]. A study further explored these potential causes by examining five types of insulin pumps available in 2020 [95]. The most common technical issues identified were related to the pod reservoir, flow alarm obstruction, or infusion set/site [94,95]. While these results may vary depending on the manufacturer, they were generally found to be the major sources of device failure [95]. Similarly, other studies have also emphasized the significance of the technology follow-up aspect, particularly highlighting the consequences of keeping infusion sets for a prolonged period, more than three days [93,96], which frequently results in catheter blockage, making up 54.3% of infusion set problems [96], and other complications also observed such as kinking, leakage, and adhesive loosening [93,96]. When diabetes devices fail to operate as intended, resulting in inadequate insulin delivery, this is reflected in the glycemic levels, as proven by a case report [97]. To elaborate, two children with T1D using CSII experienced technical issues in the system, causing detachment of the needle from the insulin infusion set and hyperglycemia [97]. This critical incident highlighted the risk that such dysfunctions may pose to patients’ glycemic management and overall health [97]. Most of the presented studies exemplify the practical issues caused by these technological systems, further complicating the seamless integration of devices into children’s and adolescents’ dynamic lifestyles to reach ideal glycemic levels.

### 2.11. Dermatological Complications

In addition to constantly tolerating the challenges and the appearance of this on-body device experience, CSII or CGM’s prolonged and minimally invasive skin penetration can be accompanied by dermatological repercussions [81,98,99,100,101,102,103,104,105]. The types of cutaneous reactions most frequently observed at the site of insertion include irritations [98], scarring [81], lipohypertrophy [81,96,99], and eczema [81,99,100]. A study examining past and recent dermatological complications in children and adolescents showed that 90% of 143 patients using CSII had previously experienced skin problems, with 63% still having ongoing issues [100]. It also showed that among 76 CGM users, 46% are currently dealing with dermatological complications as well [100]. Moreover, another observational study reported similar results, indicating that among 369 T1D patients younger than 20 years old, 91.8% experienced skin complications caused by CSII and CGM use and MDI [98]. This confirms the considerable prevalence of device-associated cutaneous side effects in the pediatric population [98], with a notable persistence observed [100]. Specifically, 81% of 138 young patients showed no improvement in their skin condition after a five-month follow-up period [101]. Furthermore, a study highlighted an elevated number of allergic contact dermatitis (ACD) cases linked to medical devices used in T1D therapy, notably among children [101]. Researchers identified isobornyl acrylate (IBOA), present in CGM and CSII adhesives, as one of the considerable causative allergens for ACD [102,103]. Thus, the skin reactions discussed are among the numerous other challenges contributing to nonadherence to medical devices in T1D, as demonstrated in a study conducted at Columbia University Irving Medical Center [104]. A group of 121 young patients with T1D using CSII and/or CGM were involved, and the results indicated that 22% interrupted their use of medical devices due to their skin complications [104]. Consequently, these dermatological side effects could substantially hinder children and adolescents from effectively controlling glucose levels using medical advances.

## 3. Solutions to Improve Therapeutic Adherence in Adolescents with T1D

Managing T1D in adolescents is a complex task, as it is considered one of the most demanding chronic diseases from a psychosocial and behavioral perspective. Routine diabetes care practice should include mental health screening of all adolescents soon after diagnosis and thereafter, with further parent/family assessment and psychological support addressing both psychological well-being and adherence issues directed to at-risk youth.

Recent studies highlight that digital health interventions can effectively support mental health and well-being in adolescents with T1D [105]. A study by Geirhos et al., 2022 [105,106] examined the effects of an emerging “internet- and mobile-based cognitive behavioral therapy” (iCBT) program called youthCOACHCD in reducing anxiety and depression in adolescents with chronic conditions such as T1D. Participants reported positive feedback with a potential reduction in psychological conditions. However, no short-term effects were observed [107]. In addition, adolescents who participated in a six-week online support intervention, “Nothing Ventured, Nothing Gained,” showed improved mental health with lower levels of depression and anxiety after three assessments (baseline, post-intervention, and at the 6-month follow-up) compared to the control group. The intervention also included sessions for improving parents’ knowledge and skills in order to facilitate T1D management and communication with their adolescents effectively, enriching family dynamics. It also facilitated a smoother transition from parent-led to adolescent-led diabetes care [107]. A 14-weekly, 1-hour session of motivational interviewing/cognitive behavior therapy (MI/CBT), clinic-based contingency management (CM), and parent-directed contingency management (CM) study for adolescents and their parents showed the importance of active engagement of both parties. Supportive family involvement showed better glycaemic outcomes, blood glucose monitoring, and HBA1c, and improved adherence to regimen practices [108]. Furthermore, the use of mobile applications like MyT1DHero observed over 12 weeks in a clinical trial involving 30 adolescents with their parents promoted better communication between both parties. Parents remained involved by receiving messages when their child entered blood glucose levels, allowing them to stay updated without creating conflict during this transitional phase to independent care [109].

It is crucial to promote diabetes education and knowledge about associated complications in order to enhance adolescents’ acceptance and awareness and to encourage treatment practices in public places, especially schools [110]. A study focussing on interventions to socially empower adolescents with T1D for improving treatment adherence also centred on educational programs, social support, and open communications with physicians, all to create effective strategies that may reduce pressure and challenges on T1D management in social settings [111].

As previously discussed, adolescents’ drive towards adventure and exploration leads to risky behaviours that hinder their commitment to T1D care [41,42,43,44,45,46,47]. Hence, a study highlighted the importance of nurses’ assistance and providing continuous education to reach effective diabetes management strategies, leading to better health outcomes in adolescents with T1D [110].

Amidst the challenges faced by adolescents, particularly inevitable ones such as the impact of puberty on T1D, self-regulation is very important to help them navigate through their condition and treatment regimen on a daily basis [112]. A study that focused on self-regulation aimed to improve 3 related factors: executive functioning, emotion regulation, and future orientation. It described exercises for memory and planning, psychological challenges, and futuristic goals, respectively, which is an approach for enhancing self-regulation, and it reported better treatment adherence to the T1D regimen [112].

Moreover, concerning financial burdens, free diabetic provisions, insulin, syringes, and glucometers, accessibility, or lower expenses could aid in improving treatment adherence [110]. Another method for addressing financial limitations would be the call for public insurance to provide coverage for continuous glucose monitoring (CGM) to allow such patients to have effective diabetic management similar to that of patients under private insurance [113]. Although adolescents encounter daily challenges in using diabetes management devices [92,93], educating patients on using technology and devices is essential to elucidate their effectiveness in controlling glycaemic levels [106]. These tools allow them to reach optimal care by staying updated on their condition and facilitating communication with healthcare providers [106]. Table 1 summarises the possible solutions to improve T1D adherence in adolescents.

## 4. Conclusions

Chronic diseases come with many responsibilities despite technologies and efforts to minimize complexity. Age plays a considerable role in this aspect, as in this case, where adolescents find it rather challenging to comprehend T1D in general, and particularly regarding its treatment regimen at their level of development. This review study revealed an extensive picture of the reality of managing T1D in adolescence daily. During this pivotal developmental stage characterized by rapid changes, patients face regimen-related barriers that prevent them from remaining within the optimal glycemic range. The multifactorial stressors, summarized in Table 2, assessed in this review mainly included physiological changes, mental health burdens, societal pressure, and the impact of medical devices on users’ lives. Most studies identified a strong correlation between treatment adherence and glycemic levels, prioritizing further understanding of adolescence. Addressing the extent to which the complex barriers impede diabetes management is crucial to reaching an effective course of treatment in this critical age group.

## Figures and Tables

**Table 1:** Possible tools for improving type 1 diabetes adherence in youth [106,107,108,109,110,111,112,113].

Possible Solutions to Improve Type 1 Diabetes Adherence in Adolescents		
MENTAL HEALTH	THERAPY-RELATED FACTORS	SOCIAL FACTORS
Acknowledge psychological factors and prioritize mental well-being. Routine mental health and behavioral screening as part of diabetes care. Psychological support, including digital health interventions.	Discussions on the transition to adult diabetes care. Support and coordination between pediatric and adult care providers. Integrate user-friendly technological solutions.	Foster peer support and social engagement. Implement peer education programs in schools about type 1 diabetes, reducing stigma. Ensure access to trained health professionals like school nurses.
**TRANSITIONING RESPONSIBILITY**	**RISK-TAKING BEHAVIOURS**	**PUBERTAL CHANGES**
Proper balance between parental involvement and adolescent autonomy. Support adolescents to take ownership of their health. Family-focused teamwork, developing shared parent-youth responsibility and strategies to avoid conflict.	Screening for alcohol, drug, or tobacco use should be included in the routine medical care for adolescents with diabetes. Manage risk-taking behaviors.	Explain that increased insulin resistance is a temporal part of puberty. Support building a powerful self-identity. Help adolescents develop healthy body image.

**Table 2:** Summarization of the most common obstacles to T1D adherence in youth [24,25,26,27,28,32,33,34,35,37,38,39,42,43,44,49,50,51,54,74,75,76,81,84,85,86,92,93,99,101].

The Most Common Barriers to Type 1 Diabetes Adherence in Adolescents		
MENTAL HEALTH	THERAPY-RELATED FACTORS	SOCIAL FACTORS
Stress/Anxiety/Depression Diabetes-related fear of hypoglycemia. Behavioral disorders. Poor acceptance of illness. Eating disorders.	Complexity of treatment. Cost of care. Poor level of diabetes education. Unsatisfactory relationship with healthcare providers. Inequalities of medication supply/services. Beliefs about medicines.	Family dynamics/relations/support. Stigma. Bullying. Social/school support. Social exclusion. Disease-related embarrassment.
**TRANSITIONING RESPONSIBILITY**	**RISK-TAKING BEHAVIOURS**	**PUBERTAL CHANGES**
Not defining the role of parents and adolescents in diabetes management. Insufficient knowledge of diabetes. Fear of being independent. Poor management strategies. Parental overcontrolling. Lack of caregiver support.	Alcohol use. Drug abuse. Smoking.	Increased insulin resistance. Weight and body image concerns. Poor sense of self-identity. Difficulties in integrating diabetes into a new lifestyle.

## Data Availability

No new data was created or analyzed in this study. Data sharing is not applicable to this article.

## References

[1] Haeusler R.A., McGraw T.E., Accili D. (2017). Biochemical and Cellular Properties of Insulin Receptor Signalling. Nat. Rev. Mol. Cell Biol..

[2] Tokarz V.L., MacDonald P.E., Klip A. (2018). The Cell Biology of Systemic Insulin Function. J. Cell Biol..

[3] Powers A.C. (2021). Type 1 Diabetes Mellitus: Much Progress, Many Opportunities. J. Clin. Investig..

[4] Cengiz E., Danne T., Ahmad T., Ayyavoo A., Beran D., Ehtisham S., Fairchild J., Jarosz-Chobot P., Ng S.M., Paterson M. (2022). Ispad Clinical Practice Consensus Guidelines 2022: Insulin Treatment in Children and Adolescents with Diabetes. Pediatr. Diabetes.

[5] Lawrence J.M., Divers J., Isom S., Saydah S., Imperatore G., Pihoker C., Marcovina S.M., Mayer-Davis E.J., Hamman R.F., Dolan L. (2021). Trends in Prevalence of Type 1 and Type 2 Diabetes in Children and Adolescents in the US, 2001–2017. JAMA.

[6] (2021). IDF Diabetes Atlas.

[7] American Diabetes Association (2009). Diagnosis and Classification of Diabetes Mellitus. Diabetes Care.

[8] American Diabetes Association (2020). Addendum. 9. Pharmacologic Approaches to Glycemic Treatment: *Standards of Medical Care in Diabetes—2020*. Diabetes Care.

[9] Herman W.H. (2015). Response to Comment on American Diabetes Association. Approaches to Glycemic Treatment. Sec. 7. In *Standards of Medical Care in Diabetes—2015*. Diabetes Care 2015;38(Suppl. 1):S41–S48. Diabetes Care.

[10] KyngÄs H.A., Kroll T., Duffy M.E. (2000). Compliance in Adolescents with Chronic Diseases: A Review. J. Adolesc. Health.

[11] Kakleas K., Kandyla B., Karayianni C., Karavanaki K. (2009). Psychosocial Problems in Adolescents with Type 1 Diabetes Mellitus. Diabetes Metab..

[12] Miller K.M., Foster N.C., Beck R.W., Bergenstal R.M., DuBose S.N., DiMeglio L.A., Maahs D.M., Tamborlane W.V. (2015). Current State of Type 1 Diabetes Treatment in the U.S.: Updated Data from the T1D Exchange Clinic Registry. Diabetes Care.

[13] Wong J.C., Foster N.C., Maahs D.M., Raghinaru D., Bergenstal R.M., Ahmann A.J., Peters A.L., Bode B.W., Aleppo G., Hirsch I.B. (2014). Real-Time Continuous Glucose Monitoring among Participants in the T1D Exchange Clinic Registry. Diabetes Care.

[14] Suris J.-C., Michaud P.-A., Viner R. (2004). The Adolescent with a Chronic Condition. Part I: Developmental Issues. Arch. Dis. Child..

[15] Herzer M., Hood K.K. (2010). Anxiety Symptoms in Adolescents with Type 1 Diabetes: Association with Blood Glucose Monitoring and Glycemic Control. J. Pediatr. Psychol..

[16] Bernstein C.M., Stockwell M.S., Gallagher M.P., Rosenthal S.L., Soren K. (2013). Mental Health Issues in Adolescents and Young Adults with Type 1 Diabetes. Clin Pediatr..

[17] Shapira A., Harrington K.R., Goethals E.R., Volkening L.K., Laffel L.M. (2021). Health-related Quality of Life in Youth with Type 1 Diabetes: Associations with Multiple Comorbidities and Mental Health Conditions. Diabet. Med..

[18] Butwicka A., Fendler W., Zalepa A., Szadkowska A., Zawodniak-Szalapska M., Gmitrowicz A., Mlynarski W. (2016). Psychiatric Disorders and Health-Related Quality of Life in Children with Type 1 Diabetes Mellitus. Psychosomatics.

[19] Bratke H., Sivertsen B. (2021). Mental and Somatic Health in University Students with Type 1 Diabetes: New Results from Diashot18, a Cross Sectional National Health and Well-Being Survey. J. Pediatr. Endocrinol. Metab..

[20] Northam E.A., Lin A., Finch S., Werther G.A., Cameron F.J. (2010). Psychosocial Well-Being and Functional Outcomes in Youth with Type 1 Diabetes 12 Years after Disease Onset. Diabetes Care.

[21] de Wit M., Gajewska K.A., Goethals E.R., McDarby V., Zhao X., Hapunda G., Delamater A.M., DiMeglio L.A. (2022). ISPAD Clinical Practice Consensus Guidelines 2022: Psychological care of children, adolescents and young adults with diabetes. Pediatr. Diabetes.

[22] Colton P., Olmsted M., Daneman D., Rydall A., Rodin G. (2004). Disturbed eating behavior and eating disorders in preteen and early teenage girls with type 1 diabetes: A case-controlled study. Diabetes Care.

[23] Anderson B.J., Holmbeck G., Iannotti R.J., McKay S.V., Lochrie A., Volkening L.K., Laffel L. (2009). Dyadic Measures of the Parent–Child Relationship during the Transition to Adolescence and Glycemic Control in Children with Type 1 Diabetes. Fam. Syst. Health.

[24] Mellin A.E. (2004). Parenting Adolescent Girls with Type 1 Diabetes: Parents’ Perspectives. J. Pediatr. Psychol..

[25] Hauser S.T., Jacobson A.M., Lavori P., Wolfsdorf J.I., Herskowitz R.D., Milley J.E., Bliss R., Wertlieb D., Stein J. (1990). Adherence among Children and Adolescents with Insulin-Dependent Diabetes Mellitus over a Four-Year Longitudinal Follow-up: II. Immediate and Long-Term Linkages with the Family Milieu. J. Pediatr. Psychol..

[26] Goethals E.R., Oris L., Soenens B., Berg C.A., Prikken S., Van Broeck N., Weets I., Casteels K., Luyckx K. (2017). Parenting and Treatment Adherence in Type 1 Diabetes throughout Adolescence and Emerging Adulthood. J. Pediatr. Psychol..

[27] Duke D.C., Geffken G.R., Lewin A.B., Williams L.B., Storch E.A., Silverstein J.H. (2008). Glycemic Control in Youth with Type 1 Diabetes: Family Predictors and Mediators. J. Pediatr. Psychol..

[28] Campbell M.S., Berg C.A., Wiebe D.J. (2019). Parental Self-Control as a Moderator of the Association between Family Conflict and Type 1 Diabetes Management. J. Pediatr. Psychol..

[29] Anderson B.J. (2004). Family Conflict and Diabetes Management in Youth: Clinical Lessons from Child Development and Diabetes Research. Diabetes Spectr..

[30] Lv W., Luo J., Long Q., Yang J., Wang X., Guo J. (2021). Factors Associated with Adherence to Self-Monitoring of Blood Glucose among Young People with Type 1 Diabetes in China: A Cross-Sectional Study. Patient Prefer. Adherence.

[31] Zhang L., Ellis D.A., Naar-King S., Moltz K., Carcone A.I., Dekelbab B. (2016). Effects of Socio-Demographic Factors on Parental Monitoring, and Regimen Adherence among Adolescents with Type 1 Diabetes: A Moderation Analysis. J. Child. Fam. Stud..

[32] Temmen C.D., Lu R., Gee B.T., Chen Z., Nansel T.R. (2022). Latent Classifications of Parental Involvement in Diabetes Management for Youth with Type 1 Diabetes: A Randomized Clinical Trial. Pediatr. Diabetes.

[33] Grey M., Jaser S.S., Whittemore R., Jeon S., Lindemann E. (2011). Coping Skills Training for Parents of Children with Type 1 Diabetes: 12-Month Outcomes. Nurs. Res..

[34] Anderson B.J., Vangsness L., Connell A., Butler D., Goebel-Fabbri A., Laffel L.M. (2002). Family Conflict, Adherence, and Glycaemic Control in Youth with Short Duration Type 1 Diabetes. Diabet. Med..

[35] Marker A.M., Noser A.E., Clements M.A., Patton S.R. (2018). Shared Responsibility for Type 1 Diabetes Care Is Associated with Glycemic Variability and Risk of Glycemic Excursions in Youth. J. Pediatr. Psychol..

[36] Commissariat P.V., Kenowitz J.R., Trast J., Heptulla R.A., Gonzalez J.S. (2016). Developing a Personal and Social Identity with Type 1 Diabetes during Adolescence: A Hypothesis Generative Study. Qual. Health Res..

[37] Pittet I., Berchtold A., Akre C., Michaud P.-A., Suris J.-C. (2010). Are Adolescents with Chronic Conditions Particularly at Risk for Bullying?. Arch. Dis. Child..

[38] Sentenac M., Gavin A., Arnaud C., Molcho M., Godeau E., Gabhainn S.N. (2011). Victims of Bullying among Students with a Disability or Chronic Illness and Their Peers: A Cross-National Study between Ireland and France. J. Adolesc. Health.

[39] de Núñez-Baila M., Gómez-Aragón A., González-López J.R. (2021). Social Support and Peer Group Integration of Adolescents with Diabetes. Int. J. Environ. Res. Public Health.

[40] Wang Y.-L., Brown S.A., Horner S.D. (2013). The School-Based Lived Experiences of Adolescents with Type 1 Diabetes. J. Nurs. Res..

[41] Hogendorf A.M., Fendler W., Sierosławski J., Bobeff K., Węgrewicz K., Malewska K.I., Przudzik M.W., Szmigiero-Kawko M., Sztangierska B., Myśliwiec M. (2017). Alcohol and Cigarette Use among Adolescents with Type 1 Diabetes. Eur. J. Pediatr..

[42] Pastor A., Conn J., MacIsaac R.J., Bonomo Y. (2020). Alcohol and illicit drug use in people with diabetes. Lancet Diabetes Endocrinol..

[43] Cameron F.J., Garvey K., Hood K.K., Acerini C.L., Codner E. (2018). ISPAD Clinical Practice Consensus Guidelines 2018: Diabetes in adolescence. Pediatr. Diabetes.

[44] Gartner A., Daniel R., Farewell D., Paranjothy S., Townson J., Gregory J.W. (2020). Demographic and socioeconomic patterns in the risk of alcohol-related hospital admission in children and young adults with childhood onset type-1 diabetes from a record-linked longitudinal population cohort study in Wales. Pediatr. Diabetes.

[45] Kinney G.L., Akturk H.K., Taylor D.D., Foster N.C., Shah V.N. (2020). Cannabis Use Is Associated With Increased Risk for Diabetic Ketoacidosis in Adults With Type 1 Diabetes: Findings From the T1D Exchange Clinic Registry. Diabetes Care.

[46] Akturk H.K., Taylor D.D., Camsari U.M., Rewers A., Kinney G.L., Shah V.N. (2019). Association between Cannabis Use and Risk for Diabetic Ketoacidosis in Adults with Type 1 Diabetes. JAMA Intern. Med..

[47] Pancer J., Dasgupta K. (2020). Effects of Cannabis Use in Youth and Young Adults with Type 1 Diabetes: The Highs, the Lows, the Don’t Knows. Can. J. Diabetes.

[48] Veldhuis J.D. (1996). Neuroendocrine Mechanisms Mediating Awakening of the Human Gonadotropic Axis in Puberty. Pediatr. Nephrol..

[49] Amiel S.A., Sherwin R.S., Simonson D.C., Lauritano A.A., Tamborlane W.V. (1986). Impaired Insulin Action in Puberty. N. Engl. J. Med..

[50] Goran M.I., Gower B.A. (2001). Longitudinal Study on Pubertal Insulin Resistance. Diabetes.

[51] Moran A., Jacobs D.R., Steinberger J., Cohen P., Hong C.-P., Prineas R., Sinaiko A.R. (2002). Association between the Insulin Resistance of Puberty and the Insulin-like Growth Factor-i/Growth Hormone Axis. J. Clin. Endocrinol. Metab..

[52] Cree-Green M., Stuppy J.J., Thurston J., Bergman B.C., Coe G.V., Baumgartner A.D., Bacon S., Scherzinger A., Pyle L., Nadeau K.J. (2018). Youth with Type 1 Diabetes Have Adipose, Hepatic, and Peripheral Insulin Resistance. J. Clin. Endocrinol. Metab..

[53] Roden M., Price T.B., Perseghin G., Petersen K.F., Rothman D.L., Cline G.W., Shulman G.I. (1996). Mechanism of Free Fatty Acid-Induced Insulin Resistance in Humans. J. Clin. Investig..

[54] Moran A., Jacobs D.R., Steinberger J., Hong C.P., Prineas R., Luepker R., Sinaiko A.R. (1999). Insulin Resistance during Puberty: Results from Clamp Studies in 357 Children. Diabetes.

[55] Saenger P. (2003). Dose Effects of Growth Hormone during Puberty. Horm. Res..

[56] Bjornstad P. (2015). Insulin Sensitivity and Complications in Type 1 Diabetes: New Insights. World J. Diabetes.

[57] Millstein R.J., Pyle L.L., Bergman B.C., Eckel R.H., Maahs D.M., Rewers M.J., Schauer I.E., Snell-Bergeon J.K. (2018). Sex-Specific Differences in Insulin Resistance in Type 1 Diabetes: The Cacti Cohort. J. Diabetes Complicat..

[58] Khadilkar A., Oza C., Mondkar S.A. (2023). Insulin Resistance in Adolescents and Youth with Type 1 Diabetes: A Review of Problems and Solutions. Clin. Med. Insights Endocrinol. Diabetes.

[59] Albertini J.-P., McMorn S.O., Chen H., Mather R.A., Valensi P. (2007). Effect of Rosiglitazone on Factors Related to Endothelial Dysfunction in Patients with Type 2 Diabetes Mellitus. Atherosclerosis.

[60] Hoffman R.P., Vicini P., Sivitz W.I., Cobelli C. (2000). Pubertal Adolescent Male-Female Differences in Insulin Sensitivity and Glucose Effectiveness Determined by the One Compartment Minimal Model. Pediatr. Res..

[61] Duca L.M., Maahs D.M., Schauer I.E., Bergman B.C., Nadeau K.J., Bjornstad P., Rewers M., Snell-Bergeon J.K. (2016). Development and Validation of a Method to Estimate Insulin Sensitivity in Patients with and without Type 1 Diabetes. J. Clin. Endocrinol. Metab..

[62] Šimonienė D., Platūkiene A., Prakapienė E., Radzevičienė L., Veličkiene D. (2020). Insulin Resistance in Type 1 Diabetes Mellitus and Its Association with Patient’s Micro- and Macrovascular Complications, Sex Hormones, and Other Clinical Data. Diabetes Ther..

[63] Santos L.C., de Cintra I., Fisberg M., Martini L.A. (2008). Body Trunk Fat and Insulin Resistance in Post-Pubertal Obese Adolescents. Sao Paulo Med. J..

[64] Mann N.P., Johnston D.I. (1984). Improvement in Metabolic Control in Diabetic Adolescents by the Use of Increased Insulin Dose. Diabetes Care.

[65] Teló G.H., Dougher C.E., Volkening L.K., Katz M.L., Laffel L.M. (2018). Predictors of Changing Insulin Dose Requirements and Glycaemic Control in Children, Adolescents and Young Adults with Type 1 Diabetes. Diabet. Med..

[66] Nansel T.R., Lipsky L.M., Liu A. (2016). Greater Diet Quality Is Associated with More Optimal Glycemic Control in a Longitudinal Study of Youth with Type 1 Diabetes. Am. J. Clin. Nutr..

[67] Mackey E.R., O’Brecht L., Holmes C.S., Jacobs M., Streisand R. (2018). Teens with Type 1 Diabetes: How Does Their Nutrition Measure Up?. J. Diabetes Res..

[68] Tikkanen-Dolenc H., Wadén J., Forsblom C., Harjutsalo V., Thorn L.M., Saraheimo M., Elonen N., Hietala K., Summanen P., Tikkanen H.O. (2020). Frequent Physical Activity Is Associated with Reduced Risk of Severe Diabetic Retinopathy in Type 1 Diabetes. Acta Diabetol..

[69] Beysel S., Unsal I.O., Kizilgul M., Caliskan M., Ucan B., Cakal E. (2018). The Effects of Metformin in Type 1 Diabetes Mellitus. BMC Endocr. Disord..

[70] Nadeau K.J., Chow K., Alam S., Lindquist K., Campbell S., McFann K., Klingensmith G., Walravens P. (2015). Effects of Low Dose Metformin in Adolescents with Type I Diabetes Mellitus: A Randomized, Double-Blinded Placebo-Controlled Study. Pediatr. Diabetes.

[71] Bjornstad P., Schäfer M., Truong U., Cree-Green M., Pyle L., Baumgartner A., Garcia Reyes Y., Maniatis A., Nayak S., Wadwa R.P. (2018). Metformin Improves Insulin Sensitivity and Vascular Health in Youth with Type 1 Diabetes Mellitus. Circulation.

[72] Libman I.M., Miller K.M., DiMeglio L.A., Bethin K.E., Katz M.L., Shah A., Simmons J.H., Haller M.J., Raman S., Tamborlane W.V. (2015). Effect of Metformin Added to Insulin on Glycemic Control among Overweight/Obese Adolescents with Type 1 Diabetes. JAMA.

[73] Hunt N.J., Lockwood G.P., Heffernan S.J., Daymond J., Ngu M., Narayanan R.K., Westwood L.J., Mohanty B., Esser L., Williams C.C. (2024). Oral Nanotherapeutic Formulation of Insulin with Reduced Episodes of Hypoglycaemia. Nat. Nanotechnol..

[74] Karges B., Schwandt A., Heidtmann B., Kordonouri O., Binder E., Schierloh U., Boettcher C., Kapellen T., Rosenbauer J., Holl R.W. (2017). Association of Insulin Pump Therapy vs Insulin Injection Therapy with Severe Hypoglycemia, Ketoacidosis, and Glycemic Control among Children, Adolescents, and Young Adults with Type 1 Diabetes. JAMA.

[75] Babiker A., Alammari N., Aljuraisi A., Alharbi R., Alqarni H., Masuadi E., Alfaraidi H. (2022). The Effectiveness of Insulin Pump Therapy versus Multiple Daily Injections in Children with Type 1 Diabetes Mellitus in a Specialized Center in Riyadh. Clin. Med. Insights Endocrinol. Diabetes.

[76] Castorani V., Favalli V., Rigamonti A., Frontino G., Di Tonno R., Morotti E., Sandullo F., Scialabba F., Arrigoni F., Dionisi B. (2023). A Comparative Study Using Insulin Pump Therapy and Continuous Glucose Monitoring in Newly Diagnosed Very Young Children with Type 1 Diabetes: It Is Possible to Bend the Curve of HbA1c. Acta Diabetol..

[77] Birkebaek N.H., Kristensen L.J., Mose A.H., Thastum M. (2014). Quality of Life in Danish Children and Adolescents with Type 1 Diabetes Treated with Continuous Subcutaneous Insulin Infusion or Multiple Daily Injections. Diabetes Res. Clin. Pract..

[78] Reid L.J., Gibb F.W., Colhoun H., Wild S.H., Strachan M.W., Madill K., Dhillon B., Forbes S. (2021). Continuous Subcutaneous Insulin Infusion Therapy Is Associated with Reduced Retinopathy Progression Compared with Multiple Daily Injections of Insulin. Diabetologia.

[79] Fu V.R., Irwine K., Browne-Cooper K., Taplin C.E., Jones T.W., Davis E.A., Abraham M.B. (2023). Outcomes and Experiences of Families with Children with Type 1 Diabetes on Insulin Pumps through Subsidised Pump Access Programs in Western Australia. Front. Endocrinol..

[80] Ritholz M.D., Smaldone A., Lee J., Castillo A., Wolpert H., Weinger K. (2007). Perceptions of Psychosocial Factors and the Insulin Pump. Diabetes Care.

[81] Binder E., Lange O., Edlinger M., Meraner D., Abt D., Moser C., Steichen E., Hofer S. (2015). Frequency of Dermatological Side Effects of Continuous Subcutaneous Insulin Infusion in Children and Adolescents with Type 1 Diabetes. Exp. Clin. Endocrinol. Diabetes.

[82] Dos Santos T.J., Dave C., MacLeish S., Wood J.R. (2021). Diabetes Technologies for Children and Adolescents with Type 1 Diabetes Are Highly Dependent on Coverage and Reimbursement: Results from a Worldwide Survey. BMJ Open Diabetes Res. Care.

[83] Bächle C., Icks A., Straßburger K., Flechtner-Mors M., Hungele A., Beyer P., Placzek K., Hermann U., Schumacher A., Freff M. (2013). Direct Diabetes-Related Costs in Young Patients with Early-Onset, Long-Lasting Type 1 Diabetes. PLoS ONE.

[84] Sumnik Z., Szypowska A., Iotova V., Bratina N., Cherubini V., Forsander G., Jali S., Raposo J.F., Stipančic G., Vazeou A. (2019). Persistent Heterogeneity in Diabetes Technology Reimbursement for Children with Type 1 Diabetes: The Sweet Perspective. Pediatr. Diabetes.

[85] O’Connor M.R., Carlin K., Coker T., Zierler B., Pihoker C. (2019). Disparities in Insulin Pump Therapy Persist in Youth with Type 1 Diabetes despite Rising Overall Pump Use Rates. J. Pediatr. Nurs..

[86] Willi S.M., Miller K.M., DiMeglio L.A., Klingensmith G.J., Simmons J.H., Tamborlane W.V., Nadeau K.J., Kittelsrud J.M., Huckfeldt P., Beck R.W. (2015). Racial-Ethnic Disparities in Management and Outcomes among Children with Type 1 Diabetes. Pediatrics.

[87] Addala A., Auzanneau M., Miller K., Maier W., Foster N., Kapellen T., Walker A., Rosenbauer J., Maahs D.M., Holl R.W. (2021). A Decade of Disparities in Diabetes Technology Use and Hba1c in Pediatric Type 1 Diabetes: A Transatlantic Comparison. Diabetes Care.

[88] Khanolkar A.R., Amin R., Taylor-Robinson D., Viner R.M., Warner J., Gevers E.F., Stephenson T. (2017). Ethnic Differences in Early Glycemic Control in Childhood-Onset Type 1 Diabetes. BMJ Open Diabetes Res. Care.

[89] Finnan M., Crespo-Ramos G., Agarwal S. (2021). 7-or: Racial/Ethnic Differences in Barriers to Type 1 Diabetes (T1D) Self-Management between Non-Hispanic Black and Hispanic Young Adults (Ya). Diabetes.

[90] Povlsen L., Olsen B., Ladelund S. (2005). Diabetes in children and adolescents from ethnic minorities: Barriers to education, treatment and good metabolic control. J. Adv. Nurs..

[91] Tanenbaum M.L., Adams R.N., Hanes S.J., Barley R.C., Miller K.M., Mulvaney S.A., Hood K.K. (2017). Optimal Use of Diabetes Devices: Clinician Perspectives on Barriers and Adherence to Device Use. J. Diabetes Sci. Technol..

[92] Schmid V., Hohberg C., Borchert M., Forst T., Pfützner A. (2010). Pilot Study for Assessment of Optimal Frequency for Changing Catheters in Insulin Pump Therapy—Trouble Starts on Day 3. J. Diabetes Sci. Technol..

[93] Ross P., Gray A., Milburn J., Kumarasamy I., Wu F., Farrand S., Armishaw J., Wiltshire E., Rayns J., Tomlinson P. (2016). Insulin Pump-Associated Adverse Events Are Common, but Not Associated with Glycemic Control, Socio-Economic Status, or Pump/Infusion Set Type. Acta Diabetol..

[94] Estock J.L., Codario R.A., Keddem S., Zupa M.F., Rodriguez K.L., DiNardo M.M. (2023). Insulin Pump-Associated Adverse Events: A Qualitative Descriptive Study of Clinical Consequences and Potential Root Causes. Diabetes Technol. Ther..

[95] Pickup J.C., Yemane N., Brackenridge A., Pender S. (2014). Nonmetabolic Complications of Continuous Subcutaneous Insulin Infusion: A Patient Survey. Diabetes Technol. Ther..

[96] Massa G., Gys I., Eyndt A.O., Wauben K., Vanoppen A. (2015). Needle Detachment from the Sure-T^®^ Infusion Set in Two Young Children with Diabetes Mellitus (DM) Treated with Continuous Subcutaneous Insulin Infusion (CSII) and Unexplained Hyperglycaemia. J. Pediatr. Endocrinol. Metab..

[97] Diedisheim M., Pecquet C., Julla J.-B., Carlier A., Potier L., Hartemann A., Jacqueminet S., Vidal-Trecan T., Gautier J.-F., Dubois Laforgue D. (2023). Prevalence and Description of the Skin Reactions Associated with Adhesives in Diabetes Technology Devices in an Adult Population: Results of the CUTADIAB Study. Diabetes Technol. Ther..

[98] Burgmann J., Biester T., Grothaus J., Kordonouri O., Ott H. (2020). Pediatric Diabetes and Skin Disease (PeDiSkin): A Cross-sectional Study in 369 Children, Adolescents and Young Adults with Type 1 Diabetes. Pediatr. Diabetes.

[99] Berg A.K., Olsen B.S., Thyssen J.P., Zachariae C., Simonsen A.B., Pilgaard K., Svensson J. (2018). High Frequencies of Dermatological Complications in Children Using Insulin Pumps or Sensors. Pediatr. Diabetes.

[100] Weng A.T., Zachariae C., Christensen K.B., Svensson J., Berg A.K. (2021). Five-Month Follow-up Shows No Improvement in Dermatological Complications in Children with Type 1 Diabetes Using Continuous Glucose Monitoring Systems and Insulin Pumps. J. Diabetes Sci. Technol..

[101] Ahrensbøll-Friis U., Simonsen A.B., Zachariae C., Thyssen J.P., Johansen J.D. (2021). Contact Dermatitis Caused by Glucose Sensors, Insulin Pumps, and Tapes: Results from a 5-year Period. Contact Dermat..

[102] Herman A., Darrigade A., de Montjoye L., Baeck M. (2020). Contact Dermatitis Caused by Glucose Sensors in Diabetic Children. Contact Dermat..

[103] Herman A., Aerts O., Baeck M., Bruze M., De Block C., Goossens A., Hamnerius N., Huygens S., Maiter D., Tennstedt D. (2017). Allergic Contact Dermatitis Caused by Isobornyl Acrylate in Freestyle^®^ Libre, a Newly Introduced Glucose Sensor. Contact Dermat..

[104] Rigo R.S., Levin L.E., Belsito D.V., Garzon M.C., Gandica R., Williams K.M. (2020). Cutaneous Reactions to Continuous Glucose Monitoring and Continuous Subcutaneous Insulin Infusion Devices in Type 1 Diabetes Mellitus. J. Diabetes Sci. Technol..

[105] Morales P., Valero-Moreno S., Pérez-Marín M. (2024). New technologies in psychological intervention for adolescents with type I diabetes: A systematic review. Curr. Psychol..

[106] Geirhos A., Domhardt M., Lunkenheimer F., Temming S., Holl R.W., Minden K., Warschburger P., Meissner T., Mueller-Stierlin A.S., Baumeister H. (2022). Feasibility and potential efficacy of a guided internet- and mobile-based CBT for adolescents and young adults with chronic medical conditions and comorbid depression or anxiety symptoms (youthCOACHCD): A randomized controlled pilot trial. BMC Pediatr..

[107] Hackworth N.J., Matthews J., Burke K., Petrovic Z., Klein B., Northam E.A., Kyrios M., Chiechomski L., Cameron F.J. (2013). Improving mental health of adolescents with Type 1 diabetes: Protocol for a randomized controlled trial of the Nothing Ventured Nothing Gained online adolescent and parenting support intervention. BMC Public Health.

[108] Stanger C., Ryan S.R., Delhey L.M., Thrailkill K., Li Z., Budney A.J. (2013). A Multicomponent Motivational Intervention to Improve Adherence Among Adolescents With Poorly Controlled Type 1 Diabetes: A Pilot Study. J. Pediatr. Psychol..

[109] Holtz B., Mitchell K.M., Holmstrom A.J., Cotten S.R., Dunneback J.K., Jimenez-Vega J., Ellis D.A., Wood M.A. (2021). An mHealth-Based Intervention for Adolescents with Type 1 Diabetes and Their Parents: Pilot Feasibility and Efficacy Single-Arm Study. JMIR mHealth uHealth.

[110] Dabas H., Sarin J., Madhu S. (2023). Insulin Adherence in Adolescents with Type 1 Diabetes Mellitus. Indian. J. Endocrinol. Metab..

[111] Salamon K.S., Hains A.A., Fleischman K.M., Davies W.H., Kichler J. (2010). Improving adherence in social situations for adolescents with type 1 diabetes mellitus (T1DM): A pilot study. Prim. Care Diabetes.

[112] Miller A.L., Lo S.L., Albright D., Lee J.M., Hunter C.M., Bauer K.W., King R., Clark K.M., Chaudhry K., Kaciroti N. (2020). Adolescent Interventions to Manage Self-Regulation in Type 1 Diabetes (AIMS-T1D): Randomized control trial study protocol. BMC Pediatr..

[113] Lee M.Y., Tanenbaum M.L., Maahs D.M., Prahalad P. (2022). Overcoming Barriers to Diabetes Technology in Youth with Type 1 Diabetes and Public Insurance: Cases and Call to Action. Case Rep. Endocrinol..

